# The relationship between internal and external factors about the outpatients’ choice of hospital: A cross‐sectional study from Jiaxing City, China

**DOI:** 10.1002/hsr2.821

**Published:** 2022-09-11

**Authors:** Mingming Yu, Guoyang Zhao, Dan Tang

**Affiliations:** ^1^ Department of Economics and Management Shanghai Technical Institute of Electronics and Information Shanghai China; ^2^ Department of Economics and Management Tongji Zhejiang College Jiaxing China

**Keywords:** behavioral decision‐making mechanism, internal and external factors, outpatients’ choice of hospital, sociodemographic and psychological characteristics

## Abstract

**Background and Aims:**

Exploring the mechanism influencing the choice of hospital among patients is important to render better care to them. The main purpose of this study is to evaluate the relationship between outpatients’ different internal factors (sociodemographic and psychological characteristics) and different external factors (provider characteristics) regarding their choice of hospital.

**Methods:**

The data obtained via questionnaire was analyzed with a linear regression model to verify the relationship between outpatients’ internal and external factors. In addition, for external factors, we built a score reflecting a comprehensive hospital's “hard power” (diagnosis and treatment technology and expertise, i.e., to say, the curative capability) and “soft power” (whether the environment for seeing a doctor is convenient and cheap, etc.) factors which influence the choice of outpatients, and the factors were given different points and weighted according to the option's order of the questionnaire.

**Results:**

We did not see evidence that internal factors such as gender, age, birthplace, and having or not having medical insurance had an effect on the comprehensive external factors of the hospital's choice (*p* > 0.05). However, statistically significant differences were found (*p* < 0.001) that outpatients who usually resided near Jiaxing valued hospitals’ “hard power” to a greater extent than did outpatients who lived in Jiaxing city, otherwise, “soft power” was prioritized. Similarly, outpatients who recognized themselves as having serious diseases valued hospitals’ “hard power” to a greater extent than those with moderate or minor diseases, otherwise, “soft power” was prioritized (*p* = 0.03).

**Conclusion:**

By enhancing the hospital's “soft power,” the managers of small hospitals could attract different outpatients from large hospitals, such as outpatients with minor or moderate diseases. Moreover, the regional health service organizations should promote the building of first‐ and second‐level hospitals near cities to retain more outpatients and to achieve outpatients’ diversion from large tertiary hospitals.

## 1 INTRODUCTION

The unbalanced allocation of medical resources was often discussed in health economics and management,^1^, ^2^, ^3^ few research considered this problem from the perspective of patients’ personal characteristics. For the same disease, different people have different behavioral cognition. Some people would choose service delivery based on healthcare providers, and others on healthcare outcomes^4^; some people would choose to go to a large hospital, and some people prefer to go to nearby small care institutions.^5^, ^6^ The relationship between patients’ personal characteristics and healthcare‐seeking behavioral decision‐making can give some reasons for the unbalanced allocation of medical resources in the region.^7^ So understanding the behavioral decision‐making mechanism about patients’ personal characteristics, especially in terms of their choice of hospital is very pertinent, and this is of much importance in giving an insight into how health facilities can improve their service delivery, improve client satisfaction and by extension ensure the equilibrium of medical resources.

The macrohealth systems are different from country to country, for instance, the cultural differences include the change in illness concepts and health behavior; the existence of a wide range of health services, both in quality and quantity; and the different sociodemographic conditions.^8^

As per China's national medical service system, hospitals are classified as primary, secondary, or tertiary based on their size and the level of health care provided.^9^ For instance, tertiary hospitals provide the highest level of treatment and specialization, an account of these institutions not only have received consistent policy attention and support through financial subsidies,^10^ talented personnel, and they have also made full use of the market economy system reforms during the past 30 years. Moreover, in China, the existing health policies have small price gaps for medical insurance reimbursement between primary, secondary, and tertiary hospitals, and still allow the patients to choose where to attend healthcare services freely.^11^, ^12^ So although the government promotes medical alliances like developed countries to encourage the first visit to primary care institutions, the effect of this is not satisfactory in practice.^13^, ^14^ As a result, large tertiary hospitals are often overcrowded and Chinese patients increasingly experience difficulties and high costs related to obtaining medical services. The above scenario prompted the researchers to explore the factors influencing the choice of hospital among patients in China.

The literature focused on the choice from western countries were at least these aspects: what characteristics of patients who actively search for providers^15^, ^16^, ^17^; why most patients are consequently unable to make a completely rational choice^15^, ^18^, ^19^; and provider characteristics do patients base their choice of hospitals on.^15^, ^20^, ^21^ Victoor et al.^15^ carried out a scoping review of 1877 publications from western countries and concluded that patients’ choices are determined by a complex interplay between patient and provider characteristics.

A variety of patient characteristics determine whether patients make choices, are willing and able to choose, and how they choose. Many studies demonstrated that choice or decision to engage or seek a particular medical channel is influenced by a variety of sociodemographic and psychological characteristics, such as age, sex, education, profession,^8^, ^22^, ^23^ and the degree of self‐recognition of the disease,^24^, ^25^ which we regarded as patients’ internal factors. Moreover, some studies also believed the choice is also based on patients’ external factors regarded as: studies have shown that nonprice and price factors play a significant role in determining the patients choice on services provision which make them be latent utility as an outcome;^4^ other studies summarized provider characteristics are structure (the availability of providers, the accessibility of the providers, the type and size of the providers, the availability/experience/quality of the staff, the organization of healthcare, the cost of treatment), process (interpersonal factors, availability of information, continuity of treatment, waiting time, and the quality of treatment), and outcome (mortality or pressure sore rates).^15^, ^26^, ^27^, ^28^

Therefore, what is the mechanism influencing the choice of hospital among ill patients in China? How about the relationship between patients’ internal and external factors in China? To provide an answer to these questions, previous studies only explored the role of a few influencing factors about the choice,^28^, ^29^, ^30^ it is necessary to construct a comprehensive analysis and verify it.

## 2 METHODS

### 2.1 Theoretical model

The theoretical model in this study was built by combining behavioral theories and a logical framework of features‐to‐labels. Behavioral theories include Andersen's behavioral model of medical service utilization (MSUM), behavioral decision theory (BDT), and prospect theory (PT). Specifically, MSUM involves systematic research, including population features, such as socioeconomic and demographic factors.^31^ Moreover, BDT posits that the emergence of any decision is inseparable from three factors: the contextual factors underlying the decision, the features of the individual's beliefs, and the individual's preference structure.^32^, ^33^ With respect to PT, introducing psychology to economics, is based on the individual's actual state of decision‐making; it focuses on the psychological reasons for their behavior.^34^

The model's other part was regarding the framework of features‐to‐labels: we built the patients’ internal factors as features, and the comprehensive factors of provider characteristics as labels, which consisted of patients’ external factors, and the main task of the research was to extract useful features and construct the mapping from features to labels.^35^

### 2.2 Selection of patient characteristics and questionnaire design

The seven features reflecting patients’ internal factors were gender (male or female), age (children or youth, middle age, or old age), profession, birthplace (Jiaxing, near Jiaxing, or distant from Jiaxing), location of residence (Jiaxing, near Jiaxing), having or not having medical insurance (yes or no) (all of the above are social‐demographic characteristics), and the degree of self‐recognition of the disease (serious, medium, or minor; a psychological feature). Among these, the personal economic factor is very important, but due to the need to respect the privacy of patients, the profession was used as a proxy (farmer, industrial worker, staff or civil servant, freelancer, or individual owner). Different professions in China represent different social classes to some degree, and there is a correlation to some extent between profession and income.^36^ In addition, the reason why the birthplace was included in the list was that there are native and nonnative residents in a city, and their lifestyles, medical insurance, and behavior characteristics are very different in China.

The next step was to design the questionnaire. According to Zhang et al.,^37^ demographic features and economic factors are the main reasons for the need for regional medical services, which are clearly manifested in outpatients’ healthcare‐seeking behaviors.^37^ In addition, the choice of a questionnaire survey in outpatient departments can better reflect the randomness and real scene of the city population than some specific communities and units. Therefore, we selected outpatients to be the respondents of the questionnaires, and the survey city was selected in Jiaxing, a representative medium‐sized city in China.^38^

The questionnaire consisted of two parts: the first part queried seven characteristics of the outpatients, while the other part addressed patients’ healthcare‐seeking behavioral decision‐making, which was a question asking the outpatients why they chose to visit the hospital we surveyed. There were eight factors they needed to consider (for each patient, chose the most important, up to four), also the outpatients were asked to rank these factors according to their perception of the importance. All the factors were named on the basis of existing literature. Among these, four factors were regarded as indicators representing the “hard power” of the hospital. In this paper, “hard power” mainly reflected the diagnosis and treatment technology and expertise, that is to say, the curative capability of the hospital; “soft power” mainly reflected whether the environment for seeing a doctor is convenient and cheap, that is, to say, the patients’ subjective feelings about the hospital they choose except the curative capability. The four factors were as follows: A, the hospital or doctors in the hospital have a high level of medical expertise; G, the hospital has advanced diagnostic equipment; F, the hospital has available renowned experts from large hospitals for consultations; and E, doctors in this hospital demonstrate good attitudes and provide detailed explanations. The A, G, F, and E were assigned 4, 3, 2, and 1 points, respectively, according to the level of diagnosis and treatment. The other four factors were regarded as indicators representing the “soft power” of the hospital, which were as follows: B, the hospital is close to home; C, it is convenient to see a doctor in the hospital; D, the proportion of medical insurance reimbursement is higher in this hospital; and H, medical treatments are cheap in this hospital. The B, C, D, and H were assigned **−**4, **−**3, **−**2, and **−**1 points, respectively, according to the level of being convenient and available. In this case, a higher score indicated that the outpatient valued “hard power” to a greater extent, while lower scores indicated that the outpatient valued “soft power” to a greater extent.

In the city, the outpatient departments we administered the surveys of questionnaires were five tertiary hospitals and one primary hospital. Outpatient visits in the five tertiary hospitals totally accounted for 79.7% of all numbers in Jiaxing, 2017.^38^ In contrast, visits to primary hospitals in Jiaxing accounted for 15.2% of the total number of outpatient visits in the city, while visits to second and township hospitals accounted for 5.1%.^38^ Based on the proportion of outpatient visits in the city, we decided to do a stratified sampling survey of 200 respondents, 168 across the 5 tertiary hospitals and 32 in a primary hospital, with the ratio of respondents from tertiary to primary hospitals being of 5.3:1 (roughly in line with the ratio of 79.7%: 15.2% = 5.2:1). Given the small proportion of ‘other’ hospitals (i.e., secondary or township hospitals), we did not include them in our research). The size of the questionnaire sample was 200 patients, calculated using the following formula: $N=Z^{2}\times \left(P\times \left(1-P\right)\right)/E^{2}=196$, where, *Z* score is 1.96 when the degree of confidence is 95%, and *P* is the probability value, generally 0.5, and E is the sampling error rate taken as 7%.^39^

To ensure that the sample was randomly selected, in a given hospital, all questionnaires were administered at the same time and the respondents were selected using a random sampling approach based on outpatient ID number, without excluding any disease. The outpatients were instructed to complete the questionnaire based on their general health‐seeking behaviors. Only three and two outpatients in the tertiary and primary hospitals, respectively, refused to participate. Thus, 195 complete questionnaires were obtained. The survey process was approved and supervised by the Health Commission of Jiaxing, and all participants signed written informed consent forms before completing the questionnaire.

### 2.3 Linear regression analysis

The outpatients chose options for why they visited the hospital from eight factors in the question, meanwhile, they were also asked to rank options based on their perceived importance. We took a weighted average of these option scores. The weighting formula used was ($\bar{M}=\sum_{i=1}^{n}W_{i}M_{i}/\sum_{i=1}^{n}W_{i}$ ^)^. $\bar{M}$ is the weighted average score, $W_{i}$ is the weight value of the *i*th option, and $M_{i}$ is the score of the *i*th option.

Accordingly, we obtained the weighted average scores of the 195 outpatients. One problem raised in our analysis was whether there were causal links between these 195 scores and the seven characteristics of the outpatients. Another problem was whether characteristics could explain the target variable, namely scores. We addressed these problems using regression techniques, and through examination of the regression equation and the correlation coefficient by the methods of *F*‐test (one‐sided) and *t*‐test (two‐sided), which is proven that whether the regression equation is significant and the correlation is close, so the conclusion has practical significance. The priori level of significance we specified is 0.05.

## 3 RESULTS

### 3.1 Sample descriptive

As shown in Table 1, among the 195 samples, except for professional farmers, whether they were having medical insurance or not, and the degree of self‐recognition of the disease was serious, the classification quantity of each characteristic sample data was generally equilibrium. However, for the total permanent population in Jiaxing, the rate of participating in Jiaxing local medical insurance has increased yearly from 53.9% in 2016 to 78.4% in 2020,^40^ so the category distribution of samples basically conformed to the actual situation.

**Table 1:** Seven characteristics of the outpatients (*N* = 195)

Variable	*n*	%	Mean	Standard deviation
Gender			0.56	0.04
Male (0)	85	43.59		
Female (1)	110	56.41		
Age			1.07	0.06
<40 (0)	54	27.69		
40–60 (1)	73	37.44		
>60 (2)	68	34.87		
Profession			1.81	0.07
Farmer (0)	18	9.23		
Work (1)	57	29.23		
Staff or civil servant (2)	64	32.82		
Freelancer or individual owner (3)	56	28.72		
Birthplace			0.87	0.06
Jiaxing (0)	82	42.05		
Near Jiaxing (1)	57	29.23		
From a distance (2)	56	28.72		
Residency location			0.27	0.03
Jiaxing (0)	143	73.33		
Near Jiaxing (1)	52	26.67		
Medical insurance			0.21	0.03
Yes (0)	155	79.49		
No (1)	40	20.51		
Degree of self‐recognition of the disease			0.71	0.05
Minor (0)	76	38.97		
Medium (1)	100	51.28		
Serious (2)	19	9.75		

The data were analyzed using Stata (V13.1). In Gender, 0 denotes male, and 1 denotes female; in age, 0 denotes <40 years old, 1 denotes 40–60 years old, and 2 denotes >60 years old; in the profession, 0, 1, 2, and 3, in turn, denote farmer, industrial worker, staff or civil servant, and freelancer or individual owner; in birthplace, 0 denotes Jiaxing, and 1 denotes near Jiaxing, and 2 denotes distant from Jiaxing; in residence location, 0 denotes Jiaxing, while 1 denotes near Jiaxing; in medical insurance, 0 denotes “yes,” and 1 denotes “no”; in the degree of self‐recognition of the disease, the 0, 1, and 2, in turn, denote minor, medium, and serious.

### 3.2 Linear regression analysis

First, we defined the regression model ($M_{i}{=\varphi +\alpha N}_{i}{+\beta O}_{i}{+\gamma P}_{i}+{\delta Q}_{i}{+\varepsilon R}_{i}{+\tau S}_{i}{+\theta T}_{i}+\mu .$_)._ M is a continuous random variable, consisting of the weighted average scores of the outpatients; *N* is gender, *O* is age, *P* is a profession, *Q* is the birthplace, *R* is residency location, *S* is having or not having medical insurance, and *T* is the degree of self‐recognition of disease, all of which are discrete random variables. The remaining letters represent parameters and *i* represents the sample order of outpatients.

The seven variables except *T* are all dummy variables, and the dummy variables were defined using Stata (V13.1). *T* is an ordinal variable with the same span across all levels, which is, therefore, treated as a scalar continuous variable.

Next, to eliminate any adverse effect of multicollinearity on the linear multivariation regression model, stepwise regression was adopted. First, Stata (V13.1) was used to perform regressions on *M* and each independent variable. *M* and *P*2 (industrial worker), *M* and *Q*1 (near Jiaxing), *M* and *Q*2 (distant), *M* and *R*, and *M* and *T* all passed the *F*‐test (one‐sided, *p* < 0.05), which showed that their relationships are significant correlation. Then, the stepwise regression command (“swreg”) in Stata (V13.1) was used to perform stepwise regression on *M* and the five variables that passed the *F*‐test.

In the process, the two independent variables *Q*1 and *Q*2 were removed in the stepwise regression and the remaining three independent variables jointly passed the *F*‐test (one‐sided, *p* < 0.001), although the dummy_*P*2's *t*‐test *p*‐value was slightly larger (two‐sided, *p* = 0.08). See the result in Table 2.

**Table 2:** The result of stepwise regression

*M*	Coefficient	Standard error	*t*	*p* > |*t*|	95% Confidence interval
Dummy_*P*2	−0.574	0.326	−1.76	0.080	−1.218 to 0.070
*R*	2.055	0.333	6.17	0.000	1.399–2.712
*T*	0.536	0.232	2.31	0.022	0.077–0.994
_cons	−0.436	0.248	−1.76	0.080	−0.924 to 0.053

*Note*: *N* = 195, probability > *F* = 0.0000, *R* ^2^ = 0.2094, adjusted *R* ^2^ = 0.1970.

Finally, considering that the error terms of the 195 outpatients would exhibit heteroscedasticity (i.e., the features of the outpatients differed greatly, such as farmer vs. worker), we used Stata (V13.1) to perform a White test on the above model. The *p*‐value of the *χ* ^2^(2) test (one‐sided) was 0.05, namely less than 0.1, thereby implying that heteroscedasticity was present. Accordingly, weighted least squares correction was executed using the wls0 command of Stata (V13.1); thus, the final result was obtained.

After the correction of heteroscedasticity, the adjusted *R* ^2^ value rose from 0.1970 to 0.2144, and the *p*‐value of the *F*‐test (one‐sided) remained < 0.001, thereby representing an improved model. However, the dummy_*P*2 failed the *t*‐test (two‐sided, *p* = 0.20). See the result in Table 3.

**Table 3:** The result of the WLS regression

*M*	Coefficient	Standard error	*t*	*p* > |*t*|	95% Confidence interval
Dummy_*P*2	−0.425	0.337	−1.26	0.201	−1.090 to 0.240
*R*	1.764	0.267	6.60	0.000	1.237–2.290
*T*	0.432	0.197	2.19	0.030	0.042–0.821
_cons	−0.264	0.279	−0.95	0.344	−0.814 to 0.286

*Note*: *N* = 195,  probability > *F* = 0.0000, *R* ^2^ = 0.2265, adjusted *R* ^2^ = 0.2144.

Abbreviation: WLS, weighted least squares.

The final linear regression model was (${\hat{M}}_{i}=-0.264+1.764R_{i}+0.432T_{i}$_)_ which meant outpatients who usually resided near Jiaxing valued hospitals’ “hard power” when making the choice of hospital to a greater extent than did outpatients who lived in Jiaxing city. Similarly, outpatients who recognized themselves as having serious diseases valued hospitals’ “hard power” to a greater extent than those with moderate or minor diseases.

## 4 DISCUSSION

The studies regarding the patients’ choice of the hospital in different countries were different. The health insurance systems in western countries (America, Europe) differ from other countries, and the access to health care may not be limited or healthcare services may be well developed.^41^ In many western countries, patients are not encouraged to actively choose their healthcare provider, and the important reasons for promoting patient choice are to reduce waiting times and to encourage competition between providers.^42^

However, in traditional societies of the developing world, the set of determinant variables for the utilization of health services seems to be more complex than in developed countries.^8^ In the past few years, much of the hospital choice literature is replete with studies from the western world. Only some efforts in recent years have been made to conduct similar empirical studies in developing countries like Africa, Asia, and the Middle East. These studies concerned the relationship between patients' sociodemographic characteristics and the choices to attend public or private hospitals and identified various factors patients consider in choosing hospitals, these factors which were observed also had differences among the various patient's sociodemographic characteristics.^8^, ^22^, ^23^, ^28^ In addition, according to these empirical analyses, many ranked the importance of various factors, yet the results were varied, and there was no obvious consistency between different studies.^22^, ^27^

In this study, we found although there are many characteristics of hospital providers, they can be integrated into hardware and software factors, respectively. After dividing the two kinds of factors, it was easier for us to find the laws between provider characteristics and patients’ characteristics of sociodemographic and psychological. Based on the behavioral theoretical model proposed previously, we used outpatients’ seven characteristics as internal factors to be featured, and the external factors reflecting healthcare provider characteristics to be labeled. These labels can be divided into two parts: “hard power” and “soft power.” We innovated to combine the two parts into a score that can show the comprehensive value of the outpatient's choice of hospital. However, most previous studies analyzed the influencing mechanisms about the choice of the hospital just for some provider characteristics they believed were important,^26^, ^27^, ^28^ and the relationship and classification of provider characteristics were not all‐around taken into account.

Regarding the results of linear regression analysis, we can see gender, age, birthplace, and medical insurance had no effect on the comprehensive external factors. It meant under the same condition, compared with residency location and the degree of self‐recognition of disease, outpatients considered the differential between “hard power” and “soft power” was not evident to males or females, different age groups, different birthplaces, and having or not having medical insurance.

Furthermore, the results mainly showed that outpatients who recognized they had serious diseases or who lived further away from healthcare facilities considered important the “hard power” that reflected the hospital's level of medical expertise. Otherwise, “soft power” was prioritized, which reflected the convenience and attainability of hospital diagnosis and treatment. To some extent, this is consistent with the PT of patients’ healthcare‐seeking behavioral decision‐making: when outpatients consider themselves to have a serious disease, decision‐making is more affected by their risk preference and they are more inclined to consider the expertise of the care facility.^34^ In contrast, outpatients who consider themselves as having a minor disease are more likely to consider other factors, such as economics and convenience of medical treatments, and their healthcare‐seeking behaviors tend to be more comprehensive and rational.^34^ This suggests that managers of small hospitals could attract different outpatients from large hospitals, such as outpatients with minor or moderate diseases, by enhancing the hospital's “soft power.”

Moreover, outpatients who currently lived further away from the hospital were more concerned about the hospital's “hard power,” indicating that their main preference was to cure disease, with less consideration of “soft power” factors. In contrast, outpatients living near the hospital paid less attention to “hard power”; “soft power” factors were their concern, given the same seriousness of diseases as outpatients who lived further away. That is, the distance one must travel to the hospital is also an important cost consideration for outpatients. This conclusion is consistent with Wu^43^ and Jiang et al.^44^ in China: the further the distance to see a doctor, the greater the extent to which patients consider the medical expertise level of hospitals. Therefore, our suggestion is to promote the building of first‐ and second‐level hospitals near cities to retain more patients and to achieve diversion.

Last, in the linear regression analysis, we also saw that profession maybe affect the outpatients’ choice when seeking medical treatments. Therefore, if a certain profession in the region is common among patients, corresponding hospitals suitable for them should be established. However, the effect in our sample did not reach significance. Future research could look at increasing the sample size or using other methods of sampling to gain more insight into the factors affecting hospital choice decisions. In addition, the adjusted *R* ^2^ value was only 0.2144 for the model that used seven outpatient characteristics to explain the target variable, which indicates that there may remain important characteristics that we did not include.

From the results above, different patients made different choices in different situations, with different methods, we still drew basic laws similar to those of other research or countries. Therefore, although there are a large number of internal and external factors of patients, studies should sum up these factors and extract useful features and construct the mapping from features to labels about the choice of hospital.

## 5 CONCLUSION

A patient's choice of hospital is determined by a complex interplay between a variety of patients' sociodemographic and provider characteristics. The results of this study revealed that outpatients who recognize they have serious diseases or who live further away from healthcare facilities would attach importance to the hospital's level of medical expertise. Otherwise, the convenience and attainability of hospitals are prioritized.

Accordingly, it provides direction that managers of small hospitals could attract different outpatients from large hospitals, such as outpatients with minor or moderate diseases, by enhancing the hospital's “soft power.” Additionally, our suggestion is to promote the building of first‐ and second‐level hospitals near cities to retain more local patients who saw a doctor in tertiary hospitals before.

## 6 LIMITATIONS

One limitation of this study is that some important characteristics like patients’ economic factors we did not include, which led to a decline in the explanatory power of our model. Another limitation is that the study has been conducted with a limited number of respondents using stratified sampling methods.

## AUTHOR CONTRIBUTIONS

**Mingming Yu**: Conceptualization; data curation; formal analysis; funding acquisition; investigation; methodology; and project administration. **Guoyang Zhao**: Data curation; formal analysis; project administration; and writing–review and editing. **Dan Tang**: Conceptualization; project administration; and writing–review and editing.

## CONFLICT OF INTEREST

The authors declare no conflict of interest.

## TRANSPARENCY STATEMENT

The lead author Mingming Yu affirms that this manuscript is an honest, accurate, and transparent account of the study being reported; that no important aspects of the study have been omitted; and that any discrepancies from the study as planned (and, if relevant, registered) have been explained.

## Data Availability

The data of the study are available by the corresponding author upon reasonable request.
